# Chronic Vulvovaginal Pain in Patients of Color: Benefits of Partner Supportiveness in Relation to Sexual Dissatisfaction and Distress

**DOI:** 10.3390/ijerph19073975

**Published:** 2022-03-27

**Authors:** Margaret Bennett-Brown, Olivia R. Adams, Jessica T. Campbell, Zoe Moscovici, Amanda N. Gesselman

**Affiliations:** 1 The Kinsey Institute, Indiana University, Bloomington, IN 47405, USA; oliadams@iu.edu (O.R.A.); jestcamp@iu.edu (J.T.C.); zomosco@iu.edu (Z.M.); agesselm@indiana.edu (A.N.G.); 2 Department of Communication Studies, Texas Tech University, Lubbock, TX 79409, USA

**Keywords:** social support, chronic vulvovaginal pain, emotional support, sexual dissatisfaction, sexual stress, partner supportiveness

## Abstract

Within the social support literature, individuals who experience chronic pain have shown many positive outcomes and benefits when receiving the appropriate level of emotional support. In the current study, individuals who experience chronic vulvovaginal pain (CVVP) were asked about their partner’s supportiveness, other sources of emotional support, and their satisfaction and stress surrounding sexual activity. The participants (*n* = 333) also identified as people of color, with a majority identifying as African American or Black (*n* = 227). The participants indicated that their partners were overall supportive of their diagnoses and found other emotional support sources through medical professionals, vulvar/vaginal pain-specific medical information websites, and family or friends. After conducting linear regressions, results showed the partner supportiveness was associated with less distress and less dissatisfaction surrounding sexual activity. Future research is suggested to further examine social support’s role for minority patients who experience chronic vulvovaginal pain.

## 1. Introduction

Intimate relationships are linked with better mental and physical well-being across the lifespan [1,2,3]. For people with chronic illness or disease in particular, intimate relationships can greatly impact their ability to self-manage their condition, illness-related quality of life, coping responses, post-traumatic stress, and growth, among many other factors [4,5,6,7]. The downstream impact of intimate relationship quality can thus be substantial, especially for partners struggling with the day-to-day manifestations of their condition or disease [8].

When the condition or disease specifically impacts sexuality, intimate partner support is especially critical. Sexual intimacy and sexual satisfaction are crucial components of relationship quality (i.e., subjective perceptions of a relationship as good or bad) [9] and are directly linked to relationship stability [10]. Longitudinal studies of married couples have shown that sexual satisfaction at time of assessment is related to later relationship satisfaction, even in studies spanning more than a decade [11]. In fact, a recent study employing machine learning to the study of over 11,000 romantic couples found that sexual satisfaction was one of the top five predictors of higher relationship quality [12]. Although scholars are in disagreement over the true direction of the association between relationship satisfaction and sexual satisfaction—meaning which distinctly drives the other—these concepts have consistently been highly correlated in cross-sectional and longitudinal research [13]. Unsurprisingly, changes to sexual intimacy are among the most predominant concerns for people undergoing treatment for breast, colon, or reproductive cancers [5,14,15]. Such changes could leave one or both partners with unfulfilled sexual needs and potentially pose a threat to relationship longevity. In the current study, we examined sexual satisfaction and distress in the context of chronic vulvovaginal pain (CVVP).

CVVP is vulvar and/or vaginal pain that is unexplained by underlying physical trauma, medications, or any known medical conditions [16,17]. This pain can occur during day-to-day mundane activities, such as sitting for long periods of time or wearing tight clothing, that can cause prolonged vulvar pressure. CVVP can also occur during menstruation with the use of menstrual products, during gynecological pelvic exams, and during sexual activity both with and without vaginal penetration [18,19]. Although prevalence estimates are based on incomplete data, around 13 million people are likely experiencing CVVP in the United States at the time of this writing [16].

Research on CVVP has shown that the pursuit of diagnosis and treatment is an arduous journey. Many participants report that healthcare providers have insisted that their pain is psychosomatic and thus not “real” [20,21,22]. Other studies have shown that people with CVVP typically have to see multiple healthcare providers before they reach one who has the expertise needed [23]. Prolonged health-related uncertainty and navigating multiple healthcare environments is likely to produce high levels of stress for people with CVVP; as with any other chronic condition, social support is essential [24].

Social support is defined as “information leading one to believe that he/she is cared for and loved, esteemed, and a member of a network of mutual obligations” ([25] p. 300). Social support generally includes the exchange of encouragement, validation, and resources [26] and is intended as a catalyst, enhancer, or buffer [27]. In particular, emotional support is defined as providing care, love, empathy, and support [26,28,29]. The receipt of emotional support has been shown to buffer stress, particularly in situations beyond a person’s control [30]. Likewise, the receipt of emotional support reduced the odds of post-traumatic stress disorder (PTSD) symptom emergence in people who had experienced a physical assault [31]. In the context of chronic diseases, including cancer, diabetes, and lupus, emotional support contributes substantially to better psychological adjustment and subsequently to better overall well-being [32,33,34]. Furthermore, for women experiencing chronic illness, emotional support received from a romantic partner has been associated with better adjustment to the illness, less occurrence of depressive symptoms, and higher levels of marital satisfaction [24].

Unlike many other chronic conditions, however, CVVP impacts not only psychological and physical well-being but romantic relationship functioning. While intimate partners are typically among the main sources of social support for adults [35], people may be reluctant to seek support from their partners when it relates to CVVP. This is because the presence of CVVP can complicate the maintenance of sexual intimacy. As previously detailed, sexual intimacy and resulting sexual satisfaction are especially important for perceptions of relationship quality (e.g., [12]) but CVVP may prevent typical engagement in sexual behaviors [36]. This may lead some people who have a CVVP condition to choose not to disclose their condition to their partner, in order to avoid their partner viewing them differently. Studies have shown that women with CVVP feel shame because their pain interferes with their ability to sexually please their partner—something that many women feel makes them incapable of being the “perfect girlfriend” or worthy romantic partner [37]. Thus, women and others may hesitate in seeking support for their CVVP from intimate partners, because doing so would communicate their ongoing discomfort with sexual activity and highlight their perceived shortcomings, which could in turn harm the relationship.

In the current descriptive study, we examined the prevalence of disclosure to romantic partners in a sample of over 300 women experiencing CVVP. For women who had disclosed their condition to their romantic partners, we further investigated the extent to which they perceived their partners as providing relevant emotional support and how that support was (or was not) associated with sexual dissatisfaction and distress. Longitudinal studies involving control groups of women without CVVP are needed to provide the most rigorous and causally informative understanding of how CVVP impacts romantic and sexual intimacy and overall well-being; however, the literature in this area is still emerging, and the existing library is small. Here, we conducted a descriptive and correlational investigation to provide needed foundational information for future hypothesis-driven work [38,39,40]. A better understanding of health-related disclosure and partner support in the context of chronic pain conditions like CVVP can help to advance research and therapeutic intervention on physical, psychological, and social well-being.

Although there is a small but active literature on the experiences of women with CVVP, the great majority of work in this area has been conducted with samples mostly comprising White women. A clear limitation of the existing knowledge base is in the experiences of people of color who have CVVP conditions. Studies examining lifetime prevalence of chronic vulvar pain in racially diverse communities have found no difference in lifetime rates for Black versus White women, but findings have suggested that Hispanic women are between 1.4 and 1.8 times more likely than women of other races/ethnicities to experience chronic vulvar pain [18,23]. Rates of lifetime prevalence may be relatively stable across racial groups, but studies have indicated that women of color have unique struggles with CVVP. One study examining racial differences in the experience of provoked vestibulodynia—pain localized at the vulvar vestibule [41] found no difference in Black versus White women’s pain ratings, but Black women had higher rates of functional impairment caused by their vestibulodynia [42]. The disparity in impairment is especially disconcerting considering the nearly twenty percent difference in diagnostic rates for Black versus White women with chronic vulvar pain (46% vs. 63%) [18].

We know of no research on CVVP in the context of romantic relationships that focused or specifically mentioned people who identify as a race or ethnicity other than White. However, work conducted with patients navigating other chronic diseases has shown the significance of sexual intimacy in the lives of women of color. For example, in a qualitative study of Black women breast cancer survivors under the age of 45, Lewis and colleagues reported that half of the sample believed that their cancer negatively affected their relationships and particularly their partner’s emotions [43]. In this study, participants identified sexual side effects as a paramount issue in their cancer prognosis. Relatedly, another study found similarly negative romantic and sexual relationship outcomes for Black, Asian, and White breast cancer survivors, but Latina survivors experienced additional challenges due to cultural conceptions of femininity and reconciling those conceptions with the mastectomies they had received [44]. Though sourced only from a very small review of the literature, these findings make clear the likely impact of sexually- or reproductively relevant health conditions on sexual intimacy for women of color. Outside of the illness context, recent work has further demonstrated the importance of sexual intimacy on these women’s romantic relationships. Townes and colleagues conducted a study of Black women’s perceptions of sex satisfaction within the context of relationships and found that nearly 80% of the sample considered aspects of sexual intimacy to be essential for relationship functioning [45]. In the current study, we expand the existing literature, focusing specifically on the experiences of women of color in our investigation of CVVP, partner support, sexual satisfaction, and distress.

The current study focused on social support, specifically aspects of emotional support, and its impact on individuals with chronic vulvovaginal pain. This study also specifically examined people of color—predominantly Black women—which was unique from previous studies on this topic that either lacked participants of color or failed to report on racial/ethnic categories at all [46,47,48,49]. In addition, this study sought only to examine the proposed associations for individuals who were partnered in long-term, committed romantic relationships. Based on previous literature, to analyze the current sample, our research questions were: (1) how emotionally supportive are the intimate partners of the participants who have disclosed their chronic vulvovaginal pain?; (2) is more support from partners associated with lower sexual dissatisfaction and/or distress?; and (3) aside from emotional support from one’s partner, what sources are the most helpful in providing emotional support to participants who experience chronic vulvovaginal pain?

## 2. Materials and Methods

### 2.1. Participants

There were 333 female participants (identified as being assigned female at birth) who had experienced chronic vulvovaginal pain (indicated by experiencing pain for at least 3 months) in the study. Most of the participants in the study were married (94.6%), with the others either in a committed romantic relationship (3.6%) or engaged (1.5%). One participant was in another committed relationship status that was not listed. A majority identified as heterosexual (97.9%), followed by bisexual (1.2%). Two participants identified as gay or lesbian, and one identified as pansexual. Most of the participants identified as Black/African American (68.2%), followed by 15.9% identifying as Asian and 6.3% as Native American; 4.8% identified as Pacific Islander, and 4.8% identified as White (in addition to identifying as Hispanic/Latinx). One participant identified as another racial identity, and one participant identified as Middle Eastern. Fifty-eight of the participants identified themselves to be Hispanic/Latinx as well as their previously selected racial identity. In terms of education level, 6.3% had less than a high school diploma, 27.3% had obtained a high school diploma, 33% had some college but no degree, 16.8% earned a vocational or technical degree, 8.1% had obtained an associate’s degree, 8.1% had obtained a bachelor’s degree, and one participant had earned a graduate or professional degree.

### 2.2. Measures

Demographics. Demographic questions were asked to obtain information about the participants’ age, gender identification, relationship status, and sexual orientation.

Partner support. Emotional supportiveness from the participant’s partner was measured with the item “How supportive was your partner’s response when you told them that you have chronic vulvovaginal pain?”, with choices ranging from (1) very unsupportive to (7) very supportive (*M* = 5.84, *SD* = 0.86).

Dissatisfaction about sex life. Dissatisfaction was measured with the item “How often do you feel dissatisfied with your sex life?” from the Female Sexual Distress Scale-Revised [50], and participants answered on a 5-point Likert type scale ranging from “never” to “always” (*M* = 3.00, *SD* = 1.03).

Distress about sex life. Stress was measured with the item “How often do you feel distressed about your sex life?” from the Female Sexual Distress Scale-Revised [50], and participants answered on a 5-point Likert type scale ranging from “never” to “always” (*M* = 2.71, *SD* = 0.84).

Emotional support sources. Participants were asked “What information source(s) is the most helpful for emotional support?” and were allowed to select up to three choices from the following options: medical professional, general medical information website (e.g., the Mayo Clinic, WebMD, etc.), vulvar/vaginal pain-specific medical information website (e.g., National Vulvodynia Association, International Society for the Study of Vulvovaginal Disease, Vaginismus.com/Hope and Her), health-related forums and social media (e.g., Reddit, Facebook, Tumblr, Instagram, etc.), friends or family members, newspapers or magazines, government health agencies (i.e., CDC), radio or podcasts, religious organizations or leaders, lifestyle websites or blogs (e.g., Refinery29, Cosmopolitan), or none of the options. They were then asked, “What information source(s) is the least helpful for emotional support?” and were allowed to choose up to three choices from the same list provided above.

### 2.3. Procedure

Participants (*n* = 671) were recruited via the Internet. Standardized messages, including a recruitment flyer, were posted on various social media sites (e.g., Twitter, Instagram) and were subsequently shared widely by other social media users. All research procedures were approved by the university’s Institutional Review Board; furthermore, this study was deemed exempt, as it presented no more than minimal risk to subjects and followed the guidelines for ethical research presented in the Belmont Report [51]. Recruitment for this study included several eligibility requirements: age of 19 to 55, no experience of perimenopause or menopause, identifying as a person of color (via selecting either a non-White race or Hispanic/Latinx ethnicity), and having experienced vulvar, vaginal, and/or pelvic pain for at least three to six months. Participants who failed the survey attention check items or the reCAPTCHA item and participants with duplicate IP addresses were also removed from the dataset to ensure data quality. In addition, this report examined only the data of eligible participants who reported being in a form of long-term, committed relationship. After data cleaning, 333 participants were included for statistical analysis.

Eligible participants reported demographics (e.g., age, gender, sex assigned at birth, sexual orientation, education level, race, ethnicity, relationships status) and whether they had been formally diagnosed with a chronic vulvovaginal or pelvic pain condition (e.g., vulvodynia, vaginismus, provoked vestibulodynia, dyspareunia, chronic pelvic pain, or another condition). Participants who did not have a formal diagnosis reported whether they suspected they had a chronic vulvovaginal or pelvic pain condition to capture individuals who had not yet sought care or who had not yet found an appropriate healthcare specialist that could provide a diagnosis. Participants also responded to ad hoc survey items regarding their information-seeking behaviors and their experiences in deciding to disclose their chronic pain to relationship partners.

These data were part of a larger study on the multifaceted experiences of people of color living with chronic vulvovaginal and/or pelvic pain. The larger study included data on a host of health and behavioral aspects of CVVP (e.g., physical pain symptomology involved in using tampons, feelings of medical mistrust) unrelated to the current study. Here we discuss only variables related to participant demographics, partnered social support, sexual dissatisfaction, distress, and other sources of support identified by participants as helpful to their experiences with CVVP.

### 2.4. Data Analysis

SPSS (IBM) version 28 was used to analyze the data. Statistical significance was determined by *p*-values less than 0.05. Descriptive statistics were run in order to answer the research questions posed in the study to determine percentages of participants. Two separate linear regression models were used to determine if partner supportiveness was associated with less sexual distress and/or less sexual dissatisfaction.

## 3. Results

To answer our proposed research questions, we examined descriptive statistics to determine how supportive intimate partners were after their partner (the participant) disclosed their chronic vulvovaginal pain. Our results showed that partners were rated as largely supportive. On a seven-point scale, only 2.5% of the sample selected the midpoint or lower, corresponding with “neither supportive or unsupportive” or worse. Furthermore, no one reported that their partner was “very unsupportive”. One in four participants (24.5%) reported that their partner was very supportive; 38.6% reported that their partner was supportive (i.e., a 6 on the scale); and 34.4% reported that their partner was somewhat supportive.

Next, a linear regression was run to examine the relationship between partner supportiveness and dissatisfaction about the participant’s sex life. The results yielded a significant negative relationship between partner supportiveness and dissatisfaction (β = −0.30, *p* < 0.001), indicating that increased partner support was associated with lower levels of dissatisfaction. Therefore, H1 was supported by the data.

Next, a linear regression was run to examine the relationship between partner supportiveness and distress about the participant’s sex life. The results yielded a significant negative relationship between partner supportiveness and distress (β = −0.17, *p* = 0.002), indicating that increased partner support was associated with lower levels of distress. Therefore, H2 was supported by the data.

Lastly, descriptive statistics were run in order to determine what sources of emotional support participants found to be most and least helpful as related to their chronic vulvovaginal pain. The most helpful emotional support sources were medical professionals (46.8%), followed by health-related forums and social media (26.6%), family members or friends (26.1%), and vulvar/vaginal pain-specific medical information websites (24.5%). The least helpful emotional support sources were none of the options provided (28.6%), followed by vulvar/vaginal pain-specific medical information websites (20.9%) and medical professionals (19.3%). All of the values for each source for both least and most helpful are reported in Table 1 and Table 2. As participants were allowed to select up to three of the choices provided, percentages did add up to over 100%.

## 4. Discussion

In the current study, we investigated the presence of social support from romantic partners for participants experiencing chronic vulvovaginal pain (CVVP). We also examined associations between that partnered support, sexual dissatisfaction, and distress in the context of this type of pain. Our results provided evidence—albeit of the cross-sectional variety—for the positive role that social support plays for individuals who experience CVVP. Although the role of a romantic partner’s social support has been explored in several other contexts, this was the first study to examine its role in individuals who experience CVVP. The current study should help provide encouragement to individuals who experience CVVP to disclose their diagnosis, as very few participants received unsupportive responses from their partners, and most experienced very positive, supportive responses.

Additionally, higher levels of partner supportiveness were associated with lower sexual dissatisfaction for the participant as well as less distress surrounding their sex life. Because of the intimate relationship that participants shared with their partners, and the difficulties CVVP may cause in the experience of sexual intimacy, it is reasonable to believe that having one’s partner being supportive would be of the utmost importance in how the participant views their sex life and sexual experiences. This finding also suggests that additional research should aim to examine disclosure strategies for individuals with CVVP, which could assist in finding the best way to discuss this sensitive topic with sexual partners. Within future studies, researchers should examine how these relationships between emotional support and sexual dissatisfaction play out for individuals who are single or in the early stages of a relationship, as singles may be more hesitant to reveal their diagnoses to sexual partners who are not consistent or new because of the lack of trust, which takes time to establish.

Beyond the role of partner support in CVVP, we also endeavored to identify the most helpful sources of emotional support for people experiencing CVVP. Two choices emerged when examining what sources of emotional support participants found the most and least helpful. These choices, for both the most and least helpful respectively, were medical professionals and vulvar/vaginal pain-specific medical information websites. This suggests that medical professionals play a key role in not only providing their patients with medical information and diagnoses but being people that patients can turn to for emotional support, specifically for individuals who experience CVVP and may not feel that others fully understand what they are experiencing. Imploring medical professionals to listen to their patients and provide patient-centered care, particularly for patients who are experiencing CVVP, may assist with their patients’ overall sexual and mental well-being. Previous research has shown that patients with chronic pain have better outcomes when their medical providers are able to use person-centered messages and develop a supportive connection with their patients [52].

In addition to medical providers, vulvar/vaginal pain-specific medical information websites were also identified as a most and least helpful source of support for many of the participants. There may be more nuanced reasons as to why some individuals with CVVP find these websites more or less helpful, such as a lack of specific information that individuals are searching for or a lack of certain desired features. Further research is needed to analyze the content of these websites in order to identify what can be improved in order to assist individuals with CVVP.

### Limitations

Though we have expanded the literature on the understanding of social support and its impact on racial and ethnic minority people with chronic vulvovaginal pain (CVVP), the current research was not without limitations. First, we relied exclusively on self-report data collected from the internet. Though it would be difficult to collect perceptions of support in the context of CVVP beyond the self-report, future research could employ more innovative research designs to ensure consistency in findings. Furthermore, online studies have their own limitations, including sample restriction due to accessibility of the internet and associated technology (e.g., financial ability to obtain home internet access, ability to use smartphones and/or computers). Online surveys also remove the researcher’s ability to control the environment in which people answer survey questions. The survey environment has the potential to affect how willing people are to be truthful in their responses to sensitive questions. For example, people may spend less time on—and subsequently, provide lower quality data for—questions related to sexuality if they are in the presence of other people who may be able to see the survey items.

Second, our sample for this particular inquiry was restricted. The overall sample from the larger study included a wider array of participants (e.g., different relationship statuses), but our specific research inquiry necessitated a focus on people in established romantic relationships. However, people with CVVP who are single or not in stable romantic relationships need to be included in future research. Work with these populations would provide insight into how their sexual and romantic desires are impacted by pain, how they pursue partners, and where they derive support from with regards to their CVVP (e.g., friends with benefits, social or medical support). Those transitioning into a romantic relationship would also provide valuable insight into how couples navigate disclosure of their condition and subsequent support dynamics.

Additionally, our participants all resided in the United States, but we did not have data on where our participants were raised (i.e., in the United States or elsewhere). This limited our knowledge of potential cultural differences. We could not ascertain the extent to which partners were willing to discuss gynecological issues with their partners—including vaginal pain—or if their cultural expectations drove their likelihood to share, or withhold, this type of information from their partners. Future work focusing on a larger population should collect information on social–environmental factors that may impact the magnitude of observable effects.

We had intended to compare participants who had been formally diagnosed with a CVVP condition with those who had not received a formal diagnosis. This type of comparison would potentially uncover differences in sexual dissatisfaction, distress, or partner support based on whether a healthcare authority had declared one’s symptoms to be reflective of an “authentic” condition. Unfortunately, only 16% (n = 54) of the sample reported not having a formal diagnosis; 68% (n = 227) of the sample did report a formal diagnosis, and an additional 16% (n = 52) did not answer this survey question. The distinct imbalance between diagnosed and undiagnosed groups made comparisons less informative than ideal. We recommend that future researchers work to collect adequate samples of both groups to be able to examine indirect or unexpected benefits associated with receiving a formal CVVP diagnosis.

We had little variance to examine with regard to differences between participants who had and had not disclosed their pain condition to their romantic partner. The great majority of our participants had disclosed their CVVP to their romantic partner. This finding was in line with prior work on the role of romantic partners in the experience of endometriosis. Although this work found a largely negative impact of endometriosis on the romantic relationship, over 90% of women in the study reported that their partners were interested in being informed of their health and the status of their condition [53], suggesting that disclosure would likely be appreciated by most partners. However, it should be noted that good communication skills are important for navigating long-term impacts of any chronic condition. An investigation of Canadian and American women with provoked vestibulodynia—a type of chronic vulvar pain—found no consistent pattern in women’s Dyadic Sexual Communication scale scores [54]. Thus, having a condition that directly impacts one’s partnered sexuality does not inherently make one better at working with a partner toward constructive solutions. Future research on partnered communication in the context of CVVP would be useful for therapeutic interventions.

Future researchers may also wish to delve into the present topic with more specificity, examining particular facets of distress or satisfaction in the realm of sexual intimacy. In particular, studies focused on partner communication in relation to feelings of shame or of guilt around not being able to “normally” engage in sex would be highly valuable for therapeutic interventions involving couples. Other covariates such as number of children, desire to have more children, and frequency of sexual intercourse before and after CVVP diagnoses may be of use to include in future studies. Alternatively, research examining nonvaginal forms of sex that couples with CVVP may utilize to navigate sexual pain while enhancing mutual satisfaction is an important avenue of future study needed to further understand the multifaceted nature of these dynamics.

Finally, the present research was cross-sectional, giving us only a snapshot of support dynamics in the context of CVVP and an inability to prove causal relationships. Future research should examine the evolution of intimacy, support, and sexual expression as it unfolds over time within the confines of romantic relationships and beyond, perhaps through the use of longitudinal studies.

## 5. Conclusions

In the present paper, we investigated the role of social support—specifically emotional support—and its impact on 333 people of color with chronic vulvovaginal pain (CVVP). Our results suggested that social support is essential for those who are navigating CVVP and that disclosing their CVVP diagnosis to partners frequently yielded positive and inherently supportive responses. Additional research could benefit from finding suitable strategies to disclose and discuss CVVP with sexual partners to encourage positive interpersonal and sexual health outcomes. Taken together, these findings highlight the importance of emotional support in the context of navigating a sexual partnership while managing CVVP and indicate potential routes to more satisfaction and less distress in the realm of sexual connection.

## Figures and Tables

**Table 1:** Percentages for most helpful emotional support sources.

Support Source	Percentage
Medical professional	46.8%
Health related forums and social media	26.6%
Family members or friends	26.1%
Vulvar/vaginal pain-specific medical information websites	24.5%
General medical information website	12.5%
Newspapers or magazines	4.1%
Government health agencies	4.1%
Radio or Podcasts	2.9%
None of these options	2.2%
Lifestyle websites or blogs	1.2%
Religious organizations or leaders	1.0%

**Table 2:** Percentages for least helpful emotional support sources.

Support Source	Percentage
None of these options	28.6%
Vulvar/vaginal pain-specific medical information websites	20.9%
Medical professional	19.3%
Health related forums and social media	11.5%
Government health agencies	11.1%
General medical information website	10.1%
Family members or friends	9.3%
Newspapers or magazines	6.8%
Radio or podcasts	4.6%
Lifestyle websites or blogs	1.2%
Religious organizations or leaders	0.8%

## Data Availability

The data presented in this study are available on request from the corresponding author. The data are not publicly available because of privacy concerns.

## References

[1] Holt-Lunstad J., Smith T.B., Layton J.B. (2010). Social relationships and mortality risk: A meta-analytic review. PLoS Med..

[2] Rohrbaugh M.J., Shoham V., Coyne J.C. (2006). Effect of marital quality on eight-year survival of patients with heart failure. Am. J. Cardiol..

[3] Uchino B.N., Cacioppo J.T., Kiecolt-Glaser J.K. (1996). The relationship between social support and physiological processes: A review with emphasis on underlying mechanisms and implications for health. Psychol. Bull..

[4] DiMatteo M.R. (2004). Social support and patient adherence to medical treatment: A meta-analysis. Health Psychol..

[5] Gesselman A.N., Bigatti S.M., Garcia J.R., Coe K., Cella D., Champion V.L. (2017). Spirituality, emotional distress, and post-traumatic growth in breast cancer survivors and their partners: An actor–partner interdependence modeling approach. Psycho-Oncol..

[6] Gesselman A.N., Wion R.K., Garcia J.R., Miller W.R. (2021). Relationship and sexual satisfaction are associated with better disease self-management in persons with epilepsy. Epilepsy Behav..

[7] Gouin J.P., Carter C.S., Pournajafi-Nazarloo H., Glaser R., Malarkey W.B., Loving T.J., Kiecolt-Glaser J.K. (2010). Marital behavior, oxytocin, vasopressin, and wound healing. Psychoneuroendocrinology.

[8] Corbin J.M., Strauss A. (1988). Unending Work and Care: Managing Chronic Illness at Home.

[9] Fletcher G.J., Simpson J.A., Thomas G. (2000). The measurement of perceived relationship quality components: A confirmatory factor analytic approach. Personal. Soc. Psychol. Bull..

[10] Sprecher S., Cate R.M. (2004). Sexual satisfaction and sexual expression as predictors of relationship satisfaction and stability. The Handbook of Sexuality in Close Relationships.

[11] Yeh H.C., Lorenz F.O., Wickrama K.A.S., Conger R.D., Elder G.H. (2006). Relationships among sexual satisfaction, marital quality, and marital instability at midlife. J. Fam. Psychol..

[12] Joel S., Eastwick P.W., Allison C.J., Arriaga X.B., Baker Z.G., Bar-Kalifa E., Wolf S. (2020). Machine learning uncovers the most robust self-report predictors of relationship quality across 43 longitudinal couples studies. Proc. Natl. Acad. Sci. USA.

[13] McNulty J.K., Wenner C.A., Fisher T.D. (2016). Longitudinal associations among relationship satisfaction, sexual satisfaction, and frequency of sex in early marriage. Arch. Sex. Behav..

[14] Albers L.F., van Belzen M.A., van Batenburg C., Engelen V., Putter H., Pelger R.C., Elzevier H.W. (2020). Discussing sexuality in cancer care: Towards personalized information for cancer patients and survivors. Supportive Care Cancer.

[15] Veneroni L., Bagliacca E.P., Sironi G., Silva M., Casanova M., Bergamaschi L., Ferrari A. (2020). Investigating sexuality in adolescents with cancer: Patients talk of their experiences. Pediatric Hematol. Oncol..

[16] Arnold L.D., Bachmann G.A., Rosen R., Rhoads G.G. (2007). Assessment of vulvodynia symptoms in a sample of US women: A prevalence survey with a nested case control study. Am. J. Obstet. Gynecol..

[17] Lahaie M.A., Boyer S.C., Amsel R., Khalifé S., Binik Y.M. (2010). Vaginismus: A review of the literature on the classification/diagnosis, etiology and treatment. Women’s Health.

[18] Harlow B.L., Stewart E.G. (2003). A population-based assessment of chronic unexplained vulvar pain: Have we underestimated the prevalence of vulvodynia?. J. Am. Med. Women’s Assoc..

[19] Macey K., Gregory A., Nunns D., das Nair R. (2015). Women’s experiences of using vaginal trainers (dilators) to treat vaginal penetration difficulties diagnosed as vaginismus: A qualitative interview study. BMC Women’s Health.

[20] Shallcross R., Dickson J.M., Nunns D., Taylor K., Kiemle G. (2019). Women’s experiences of vulvodynia: An interpretative phenomenological analysis of the journey toward diagnosis. Arch. Sex. Behav..

[21] Sadownik L.A., Seal B.N., Brotto L.A. (2012). Provoked vestibulodynia: A qualitative exploration of women’s experiences. Br. Columbia Med. J..

[22] Ogden J., Ward E. (1995). Help-seeking behaviour in sufferers of vaginismus. Sex. Marital Ther..

[23] Harlow B.L., Kunitz C.G., Nguyen R.H., Rydell S.A., Turner R.M., MacLehose R.F. (2014). Prevalence of symptoms consistent with a diagnosis of vulvodynia: Population-based estimates from 2 geographic regions. Am. J. Obstet. Gynecol..

[24] Primomo J., Yates B.C., Woods N.F. (1990). Social support for women during chronic illness: The relationship among sources and types to adjustment. Res. Nurs. Health.

[25] Cobb S. (1976). Social support as a moderator of life stress. Psychosom. Med..

[26] House J.S. (1981). Work Stress, and Social Support.

[27] Bavik Y.L., Shaw J.D., Wang X.H. (2020). Social support: Multidisciplinary review, synthesis, and future agenda. Acad. Manag. Ann..

[28] Krause N. (1987). Understanding the stress process: Linking social support with locus of control beliefs. J. Gerontol..

[29] Weinert C. (1987). A social support measure: PRQ85. Nurs. Res..

[30] Cohen S., Spacapan S., Oskamp S. (1988). Perceived stress in a probability sample of the United States. The Social Psychology of Health.

[31] Johansen V.A., Milde A.M., Nilsen R.M., Breivik K., Nordanger D.Ø., Stormark K.M., Weisæth L. (2020). The relationship between perceived social support and PTSD symptoms after exposure to physical assault: An 8 years longitudinal study. J. Interpers. Violence.

[32] Gallant M.P. (2003). The influence of social support on chronic illness self-management: A review and directions for research. Health Educ. Behav..

[33] Manne S.L., Kashy D.A., Kissane D.W., Ozga M., Virtue S.M., Heckman C.J. (2019). The course and predictors of perceived unsupportive responses by family and friends among women newly diagnosed with gynecological cancers. Transl. Behav. Med..

[34] Rutter S.J., Kiemle G. (2015). Exploring the social and interpersonal experiences of South Asian women with a diagnosis of Systemic Lupus Erythematosus. Psychol. Health.

[35] Florian V., Mikulincer M., Bucholtz I. (1995). Effects of adult attachment style on the perception and search for social support. J. Psychol..

[36] Bergeron S., Likes W.M., Steben M. (2014). Psychosexual aspects of vulvovaginal pain. Best Pract. Res. Clin. Obstet. Gynaecol..

[37] Elmerstig E., Wijma B., Berterö C. (2008). Why do young women continue to have sexual intercourse despite pain?. J. Adolesc. Health.

[38] Boyle P. (1996). Continual importance of descriptive epidemiology in cancer research. Ann. Oncol. Off. J. Eur. Soc. Med. Oncol..

[39] Grimes D.A., Schulz K.F. (2002). Descriptive studies: What they can and cannot do. Lancet.

[40] Naito M. (2014). Utilization and application of public health data in descriptive epidemiology. J. Epidemiol..

[41] Amalraj P., Kelly S., Bachmann G.A. (2009). Historical perspective of vulvodynia. Female Sexual Pain Disorders.

[42] Brown C., Bachmann G.A., Wan J., Foster D. (2016). Pain rating in women with provoked vestibulodynia: Evaluating influence of race. J. Women’s Health.

[43] Lewis P.E., Sheng M., Rhodes M.M., Jackson K.E., Schover L.R. (2012). Psychosocial concerns of young African American breast cancer survivors. J. Psychosoc. Oncol..

[44] Ashing-Giwa K.T., Padilla G., Tejero J., Kraemer J., Wright K., Coscarelli A., Hills D. (2004). Understanding the breast cancer experience of women: A qualitative study of African American, Asian American, Latina and Caucasian cancer survivors. Psycho-Oncol. J. Psychol. Soc. Behav. Dimens. Cancer.

[45] Townes A., Herbenick D., Dodge B., Rosenberg M. (2020). Sexual variety and sexual satisfaction in Black women: Findings from a US Probability sample of women aged 18 to 83. J. Black Sex. Relatsh..

[46] Labuski C.M. (2017). A black and white issue? Learning to see the intersectional and racialized dimensions of gynecological pain. Soc. Theory Health.

[47] Marriott C., Thompson A.R. (2008). Managing threats to femininity: Personal and interpersonal experience of living with vulval pain. Psychol. Health.

[48] Brotto L.A., Basson R., Carlson M., Zhu C. (2013). Impact of an integrated mindfulness and cognitive behavioural treatment for provoked vestibulodynia (IMPROVED): A qualitative study. Sex. Relatsh. Ther..

[49] Reissing E.D., Binik Y.M., Khalifé S., Cohen D., Amsel R. (2004). Vaginal spasm, pain, and behavior: An empirical investigation of the diagnosis of vaginismus. Arch. Sex. Behav..

[50] DeRogatis L., Clayton A., Lewis-D’Agostino D., Wunderlich G., Fu Y. (2008). Validation of the female sexual distress scale-revised for assessing distress in women with hypoactive sexual desire disorder. J. Sex. Med..

[51] United States. National Commission for the Protection of Human Subjects of Biomedical, Behavioral Research (1978). The Belmont Report: Ethical Principles and Guidelines for the Protection of Human Subjects of Research.

[52] Thórarinsdóttir K., Kristjánsson K., Gunnarsdóttir T.J., Björnsdóttir K. (2019). Facilitation of a person-centered approach in health assessment of patients with chronic pain: An ethnographic study. Qual. Health Res..

[53] Facchin F., Buggio L., Vercellini P., Frassineti A., Beltrami S., Saita E. (2021). Quality of intimate relationships, dyadic coping, and psychological health in women with endometriosis: Results from an online survey. J. Psychosom. Res..

[54] Rancourt K.M., Rosen N.O., Bergeron S., Nealis L.J. (2016). Talking about sex when sex is painful: Dyadic sexual communication is associated with women’s pain, and couples’ sexual and psychological outcomes in provoked vestibulodynia. Arch. Sex. Behav..

